# Multiplexed selectivity screening of anti-GPCR antibodies

**DOI:** 10.1126/sciadv.adf9297

**Published:** 2023-05-03

**Authors:** Leo Dahl, Ilana B. Kotliar, Annika Bendes, Tea Dodig-Crnković, Samuel Fromm, Arne Elofsson, Mathias Uhlén, Thomas P. Sakmar, Jochen M. Schwenk

**Affiliations:** ^1^ Science for Life Laboratory, School of Engineering Sciences in Chemistry, Biotechnology and Health, KTH Royal Institute of Technology, 171 65 Solna, Sweden.; ^2^ Laboratory of Chemical Biology and Signal Transduction, The Rockefeller University, 1230 York Ave., New York, NY 10065, USA.; ^3^ Tri-Institutional PhD Program in Chemical Biology, New York, NY 10065, USA.; ^4^ Science for Life Laboratory, Department of Biochemistry and Biophysics, Stockholm University, 171 65 Solna, Sweden.; ^5^ Department of Neurobiology, Care Sciences and Society, Division of Neurogeriatrics, Karolinska Institutet, 171 64 Solna, Sweden.

## Abstract

G protein–coupled receptors (GPCRs) control critical cellular signaling pathways. Therapeutic agents including anti-GPCR antibodies (Abs) are being developed to modulate GPCR function. However, validating the selectivity of anti-GPCR Abs is challenging because of sequence similarities among individual receptors within GPCR subfamilies. To address this challenge, we developed a multiplexed immunoassay to test >400 anti-GPCR Abs from the Human Protein Atlas targeting a customized library of 215 expressed and solubilized GPCRs representing all GPCR subfamilies. We found that ~61% of Abs tested were selective for their intended target, ~11% bound off-target, and ~28% did not bind to any GPCR. Antigens of on-target Abs were, on average, significantly longer, more disordered, and less likely to be buried in the interior of the GPCR protein than the other Abs. These results provide important insights into the immunogenicity of GPCR epitopes and form a basis for designing therapeutic Abs and for detecting pathological auto-Abs against GPCRs.

## Teaser

A multiplexed library-to-library approach determines the selectivity of 400 antibodies against 200 solubilized GPCR receptors.

## INTRODUCTION

The superfamily of heterotrimeric guanine-nucleotide-binding regulatory protein (G protein)–coupled receptors (GPCRs) comprises ~750 membrane proteins that mediate signaling pathways for many aspects of cellular physiology and intercellular communication. GPCRs are also targets for ~30% of approved therapeutic drugs (*1*, *2*). Disruptions in GPCR function or signaling contribute to the pathophysiology of numerous disease states. Although GPCRs are one of the most targeted protein families in pharmaceutical drug development, robust and high-throughput methods to study these dynamic membrane proteins remain limited (*3*).

Anti-GPCR antibodies (Abs) have emerged as a key enabling tool in many bioanalytical methods to study the biology and molecular pharmacology of GPCRs. However, in recent years, especially in the context of proteomics studies, the widespread or indiscriminate use of Abs without their proper independent validation has been challenged (*4*). The research community has called for an end to using unvalidated Abs as an off-the-shelf solution to study human biology (*5*). The result has been the recent creation of guidelines for Ab validation (*6*) and database registration to track Ab usage across different methods and sample types (*7*). A key outcome of these efforts was recognizing that achieving better data from immunocapture assays requires defining Ab performance and utility according to sample type, target preparation, and analytical platform (*8*). Therefore, dedicated and systematic Ab validation efforts have been conducted for several technology platforms and assay formats, including immunohistochemistry (IHC) of tissue samples (*9*), immunofluorescence (IF) assays of cells (*10*, *11*), and immunoblots of samples derived from cells (*12*, *13*) and samples of soluble proteins found in blood (*14*). Defining selective Abs validated for specific applications also facilitates the identification of specific Ab epitopes to measure possible effects from drugs developed against a GPCR target of interest.

To evaluate the selectivity and potential utility of existing anti-GPCR Abs, we established a multiplexed approach in which we tested a large library of anti-GPCR Abs in parallel against a large library of engineered GPCRs. To do this, we build upon a previous proof-of-concept study that used a suspension bead array (SBA) assay and a set of heterologously expressed and solubilized GPCR constructs that included genetically encoded monoclonal Ab (mAb) epitope tags (*15*). Each multiplexed SBA assay exposes all Abs to one GPCR at a time. Each GPCR thereby serves as the on-target of a subset of the Abs, while simultaneously serving as a probable off-target for all the other Abs in the SBA. With the objective to challenge the selectivity of each Ab, we increase the likelihood of off-target GPCR recognition by presenting overexpressed off-targets in the absence of the intended GPCR. We found that 248 of 407 anti-GPCR Abs tested against a library of 215 GPCRs were selective for their intended targets. To provide a transparent and convenient access to our dataset, we present a web-based platform that enables data browsing and analysis. Our results provide insights concerning the immunogenicity of GPCRs and for the design and application of anti-GPCR Abs as tool reagents and therapeutic agents.

## RESULTS

**Fig. 1.**
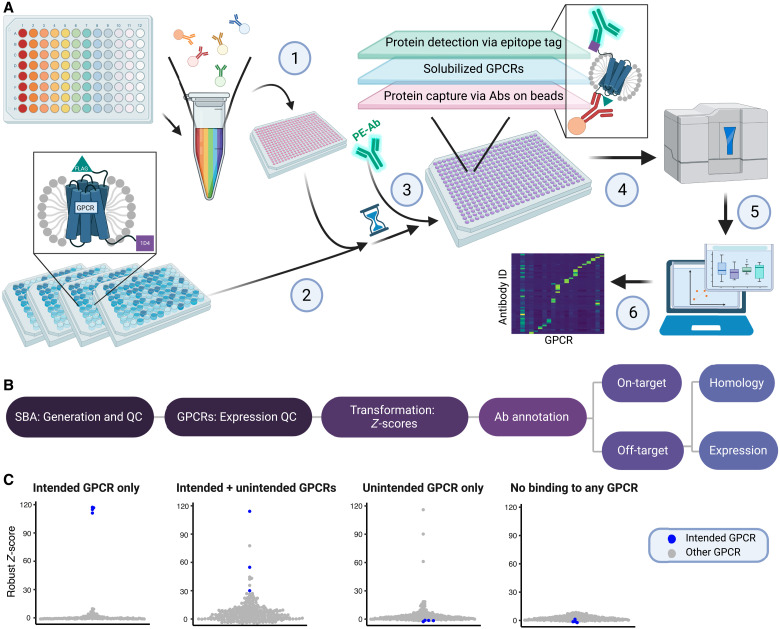
Experimental workflow and data analysis. (**A**) Abs grouped by the phylogenetic subfamily of their GPCR target were coupled to unique color-coded beads and pooled to generate subfamily-specific SBAs (1). Dual epitope-tagged GPCRs were expressed in Expi293F cells, and the membranes were solubilized in detergent, resulting in heterogeneous mixtures of solubilized membrane proteins including the GPCRs. Total protein concentration was normalized across lysates, and aliquots were incubated with the SBAs (2). PE-conjugated anti-1D4 mAb was added to detect the GPCRs captured by the bead-coupled Abs (3). A Luminex FLEXMAP 3D instrument was used to report the data (4). The data were then processed and integrated into an interactive web interface (5). The selectivity of the Abs was determined using Ab-specific thresholds (6). (**B**) Flowchart of data analysis. Data generated as described in (A) were subject to QC testing for SBA generation and GPCR expression. Next, the data were scaled and centered. Abs were then annotated as binding “on-target” or off-target, and the latter were further subclassified by the proposed cause of off-target binding. (**C**) Schematic of data visualization. Each example beeswarm plot represents a single Ab, and each dot represents a single cell-based sample. Blue dots represent samples that ectopically express the target GPCR. Gray dots represent samples that ectopically express GPCRs other than the target. The plots enable quantitative identification of four types of Ab binding behavior. Abs binding only the intended GPCR are considered validated. Abs binding intended and additional unintended GPCR targets or unintended GPCRs only are analyzed further to identify potential causes for off-target binding. Abs not binding to any target are considered not validated. Created in BioRender.com.

We established a multiplexed framework to interrogate the selectivity of anti-GPCR Abs. The workflow incorporates parallel expression of GPCRs with multiplexed protein capture assays. We ensure the detection of the immunocaptured proteins with the engineered epitope tags that allow us to determine the relative quantities of the GPCRs in solution. We then integrate the data into a web-based interface to browse the selectivity of each Ab and GPCR (Fig. 1).

We tested 407 polyclonal Abs (pAbs), of which 399 Abs were developed in the Human Protein Atlas (HPA) project and eight Abs were from other commercial sources (CABs). The HPA Abs were raised against recombinant protein fragments of 50 to 150 amino acids in length, which were selected using a bioinformatic algorithm (*16*). The approach selects unique primary structures and avoids transmembrane regions and amino acid sequences with homology to any of the other 20,000 protein-encoding gene products. Each HPA Ab was affinity purified on its respective antigen and then tested in a pipeline (*17*). Assays of the HPA pipeline include initial Ab binding assays on protein arrays built on 384 recombinant antigens (*18*) and immunoblots with six different types of samples, including tissue, cell, and plasma lysates (*19*). The primary utility of HPA Abs is to map the expression of the human protein across tissues and cells (*20*, *21*). The tissues are interrogated as sections of paraffin-embedded tissues, while the fixated cells are analyzed for the subcellular location of the protein targets by IF assay.

To generate our GPCR library, we ectopically expressed each of the 215 dual epitope–tagged GPCRs in Expi293F cells, which are suspension-adapted human embryonic kidney (HEK) 293T cells (fig. S1). Each GPCR construct includes an N-terminal FLAG tag and a C-terminal 1D4 tag, except for four GPCRs belonging to the Frizzed (FZD) subfamily, which have an N-terminal HA (hemagglutinin) epitope tag and a C-terminal 1D4 epitope tag. The tags are used to compare the quantities of receptors added to the assay and as a detection system that is common for all GPCRs. To harvest the receptors, we solubilized membranes from Expi293F cells with a buffer containing dodecyl maltoside (DM; full name *n*-dodecyl-β-d-maltoside) detergent. The detergent micelles that were generated contain a heterogeneous mixture, including the overexpressed GPCRs. These samples were then flash-frozen and thawed only shortly before the analyses.

**Fig. 2.**
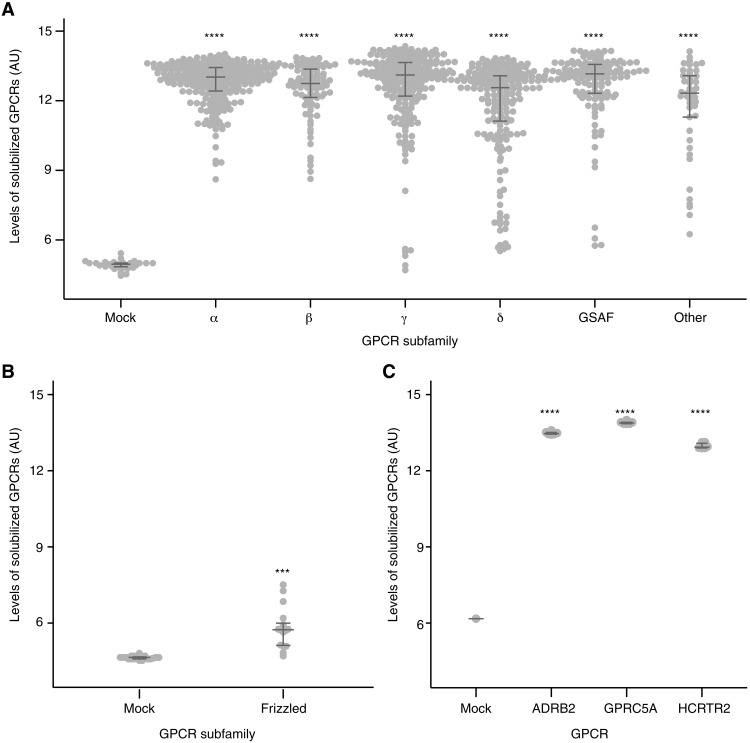
Quantification of overexpressed and solubilized GPCRs by phylogenetic group. (**A**) Relative quantities of solubilized dual epitope–tagged GPCRs were determined by SBA immunoassay. Here, FLAG was used for capture, and 1D4 was used for detection. GPCRs are grouped by subfamily. (**B**) Relative quantities of solubilized dual epitope–tagged GPCRs in the frizzled subfamily. Here, HA was used for capture, and 1D4 was used for detection. (**C**) Representative examples of GPCR expression and solubilization reproducibility. GPCRs selected from three different subfamilies were expressed in biological triplicate and quantified in technical duplicates (*N* = 6). Quantification was carried out as described in (A). Significance was determined by a one-way analysis of variance (ANOVA) (with *P* < 0.05) followed by Dunnett’s multiple comparison tests to mock. ****P < 0.0001 and ***P < 0.001. Sample sizes and *P* values are listed in table S2. Horizontal bars represent the medians of each group with the 25th and 75th percentiles. All relative levels of solubilized GPCRs were derived from MFI data and plotted here as log_2_-transformed values of arbitrary units (AU).

To create SBAs, we covalently coupled each Ab to a distinct population of color-coded magnetic beads. Abs for a specific GPCR subfamily were then pooled into subfamily-specific SBAs (Fig. 1A and table S1). The six subfamily groupings of the SBA correspond to the GRAFS classification system: rhodopsin (divided into α, β, γ, and δ), “other” and glutamate, adhesion, secretin, and FZD (GSAF) combined into one group. We chose this targeted design to investigate the Abs’ ability to differentiate between highly similar membrane proteins rather than to study a global selectivity toward a broader range of diverse receptors. Each SBA also contains Abs against the engineered epitope tags to determine the expression of the GPCRs in each assay. Because each GPCR construct contains the same pair of epitope tags, we compared the relative abundance of each GPCR across replicated preparations within each GPCR subfamily via capture of the FLAG tag and detection of the 1D4 tag, followed by a read-out in a flow cytometer (Figs. 1A and 2, A and B). We confirmed the agreement in expression between biological and technical replicates for three selected GPCRs from different phylogenetic subfamilies **(**Fig. 2C). For the selectivity screen, we used one technical replicate of four biological replicates for each GPCR-containing sample. Technical duplicates were included for a subset of GPCRs chosen at random. An essential aspect of the experimental design was to test many Abs in parallel for all six GPCR subfamilies. With this design, each Ab is exposed to its intended and overexpressed on-target GPCR and all other overexpressed family members, representing the most probable off-target GPCRs. While the primary goal is to assure that each Ab detects its intended target, the utility of each Ab increases if it does not bind to any other overexpressed off-target receptors. Therefore, with the multiplexed protein capture assays, we can determine which Abs are selective in recognizing only intended solubilized GPCR targets.

To enable a systematic assessment of Ab selectivity data at scale, we subjected the median fluorescence intensity (MFI) levels recorded per bead population and sample to several quality control (QC) steps to ensure successful Ab-bead coupling and to quantify the amounts of solubilized GPCRs added to the assay (Fig. 1B). We then scaled, centered, and transformed the raw data into *Z*-scores and robust *Z*-scores (R.*Z*-scores) for each anti-GPCR Ab separately. Last, we annotated each Ab according to its selectivity threshold and its exhibited binding to GPCRs, representing on- and off-target events. For off-target events, we conducted further analyses that considered sequence homology and expression levels as possible causes of the cross-reactivity. We visualized the performance of each Ab in beeswarm plots with R.*Z*-scores (for examples, see Fig. 1C). These examples illustrate each of the four Ab annotations: on-target, co-target, off-target, or no-target. The on-target category is defined by the data from Abs that surpass the selectivity threshold only in samples containing the intended GPCR target. The co-target category includes Abs that bind to both intended and unintended GPCR targets. They exhibit selectivity scores above the threshold in samples containing the intended GPCR and those containing an unintended GPCR(s). Co-targeting Abs could cross-react with more than one unintended GPCR. The category of off-target Abs includes those binding to only unintended GPCR(s) above the selectivity threshold. In the no-target category, the Abs did not reveal binding above the selectivity threshold for any of the tested GPCR-containing lysates. We provide the beeswarm plots and other criteria for the investigated GPCRs and tested Abs in detail in a browsable interface (Shiny app).

### Discovery of selective GPCR Abs

Our study investigated the selective binding of 407 Abs against 205 GPCRs. Although there were 215 GPCRs in the library, Abs chosen against 10 GPCRs did not pass QC for the assay. These 10 GPCRs were used as off-target candidates only.

First, we checked the relative expression of each GPCR subfamily member compared with the negative control (mock). As shown in Fig. 2 (A and B) and Table 1, most GPCRs, particularly those from the rhodopsin α and β subfamilies, were present in sufficient amounts. Only a handful of solubilized GPCRs from the rhodopsin γ and δ subfamilies, the GSAF group and the other group were detected at lower levels. The GPCRs belonging to the small FZD subfamily within the GSAF group expressed at the lowest efficiency. Considering the levels of GPCRs allowed us to judge the capability of anti-GPCR Abs for selective or cross-reactive binding.

**Table 1.** GPCR expression and recognition. A per subfamily summary of GPCRs (number of members and number expressed) is given. The total number of generated GPCRs, the percentage of GPCRs with evidence of expression (determined via the epitope tags), and the percentage of expressed GPCRs recognized by the tested anti-GPCR Abs. The latter category is limited to only GPCRs targeted by at least one Ab.

Subfamily	GPCRs (*N*)	Expressed (%)	Recognized (%)
Rhodopsin (α)	59	93.2	90.8
Rhodopsin (β)	21	85.7	78.3
Rhodopsin (γ)	51	86.3	85.9
Rhodopsin (δ)	46	73.9	71.7
GSAF	29	86.2	92.5
Other	9	66.7	60.0
Total	215	84.7	72.0

**Fig. 3.**
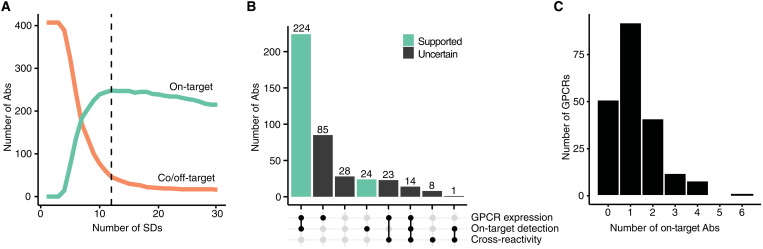
Ab selectivity threshold and summary statistics. (**A**) Data-driven selection of a hit threshold for defining Ab selectivity. The plot shows the theoretical number of Abs that would be categorized as on-target (green) and Abs that exhibit cross-reactivity (binding of unintended GPCRs, orange) as a function of SDs from the population peak. The threshold was selected to align with the plateau and corresponds to 12 SDs (vertical dashed line). (**B**) The numbers of Abs fall into different categories based on evidence of GPCR expression, target detection, and cross-reactivity. Green bars indicate Abs that captured only the intended GPCR target, irrespective of the level of GPCR expression. (**C**) Histogram showing the distribution of the number of validated Abs per GPCRs.

To determine an appropriate cutoff for defining Ab selectivity, we constructed a model to test how many Abs would pass and recognize their intended GPCR or fail because of insufficient target enrichment. We used R.*Z*-scores and the density peaks of the population data obtained per Ab and applied a function of SDs to determine the global selectivity threshold (Fig. 3A). We selected a threshold of 12 SDs over the density peak, as this SD level coincided with the peak in on-target recognitions and the beginning of the plateaus for the on- and off-target criteria. The threshold level was specific for each Ab and ranged from a R.*Z*-score of 8.7 to 195.7 (mean, 20.5). Applying these Ab-specific thresholds, we annotated each Ab for binding to the on-target GPCR, binding to the on-target and any off-target GPCR(s), detecting only an off-target GPCR, or not being able to detect any GPCR targets (Table 2). To classify an Ab as exhibiting on-target binding, the mean R.*Z*-scores obtained from the samples expressing the intended target GPCR were required to be above the cutoff. Conversely, any “off-target” data point value above the threshold resulted in an annotation as a “cross-reactive” Ab.

**Table 2.** Anti-GPCR Ab performance. A summary, per subfamily, of anti-GPCR Ab performance. The numbers of Abs and target specificity percentages per subfamily of target GPCRs are listed. On-target Abs are target-specific without cross-reactivity toward unintended GPCRs. Co-target Abs recognize both intended and unintended GPCRs. Off-target Abs recognize only unintended GPCRs. Abs with no target did not detect anything above the threshold.

Subfamily	Abs (*N*)	On-target (%)	Co-target (%)	Off-target (%)	No target (%)
Rhodopsin (α)	124	74.2	1.6	8.9	15.3
Rhodopsin (β)	54	59.3	1.9	14.8	24.1
Rhodopsin (γ)	98	54.1	5.1	4.1	36.7
Rhodopsin (δ)	72	54.2	2.8	4.2	38.9
GSAF	44	63.6	2.3	4.5	29.5
Other	15	26.7	26.7	20.0	26.7
Total	407	60.9	3.7	7.6	27.8

We found that 61% (248 of 407) of Abs tested recognized only their intended GPCR. These Abs did not exhibit any off-target enrichment above the selectivity threshold. Of the remaining 159 Abs, 9% (15 of 159) of Abs enriched the intended target and at least one off-target. Another 20% (31 of 159) of remaining Abs bound only an unintended target(s), while 71% (113 of 159) did not enrich any target above the cutoff (Fig. 3B). The 248 highly selective Abs correspond to 154 unique GPCR targets. Many GPCRs had two or more validated Abs and, in some cases, up to six Abs (Fig. 3C). Of the 407 Abs tested, we also tested 8 binders developed by sources other than the HPA. Of these eight CABs, four Abs were highly selective, corresponding to four unique GPCR targets.

To test whether any of these observations were linked to a specific subfamily, we determined the success rates of GPCR expression and recognition (Table 2, Fig. 2, and table S2). Of the 215 GPCRs, 182 (84.7%) passed the expression threshold, and the success rate for expressing GPCRs was the highest for the rhodopsin α subfamily (93.2%) and the lowest for the rhodopsin δ subfamily (73.9%). Considering only those GPCRs deemed to be expressed in statistically sufficient quantities and excluding the smallest subfamily of other GPCRs, the chances of detecting a GPCR ranged from 71.7 to 92.5%. Successful expression of the GPCRs increased the likelihood of finding on-target Abs.

### Analysis of paired Abs and different GPCR epitopes

One valuable approach to validate Abs is to compare several paired Abs that bind to different epitope regions on a common target. Among the 205 GPCRs targeted by Abs, 116 (57%) were targeted by two or more paired Abs, enabling us to compare binding characteristics to a GPCR and present different scenarios for selective and nonselective recognition of solubilized receptors. Overall, 62 of 116 (53%) of GPCRs with paired Abs were recognized by ≥2 Abs. We evaluated agreement between paired Abs using Pearson correlation and found it to be elevated (mean of *r* = 0.71; median of *r* = 0.89).

**Fig. 4.**
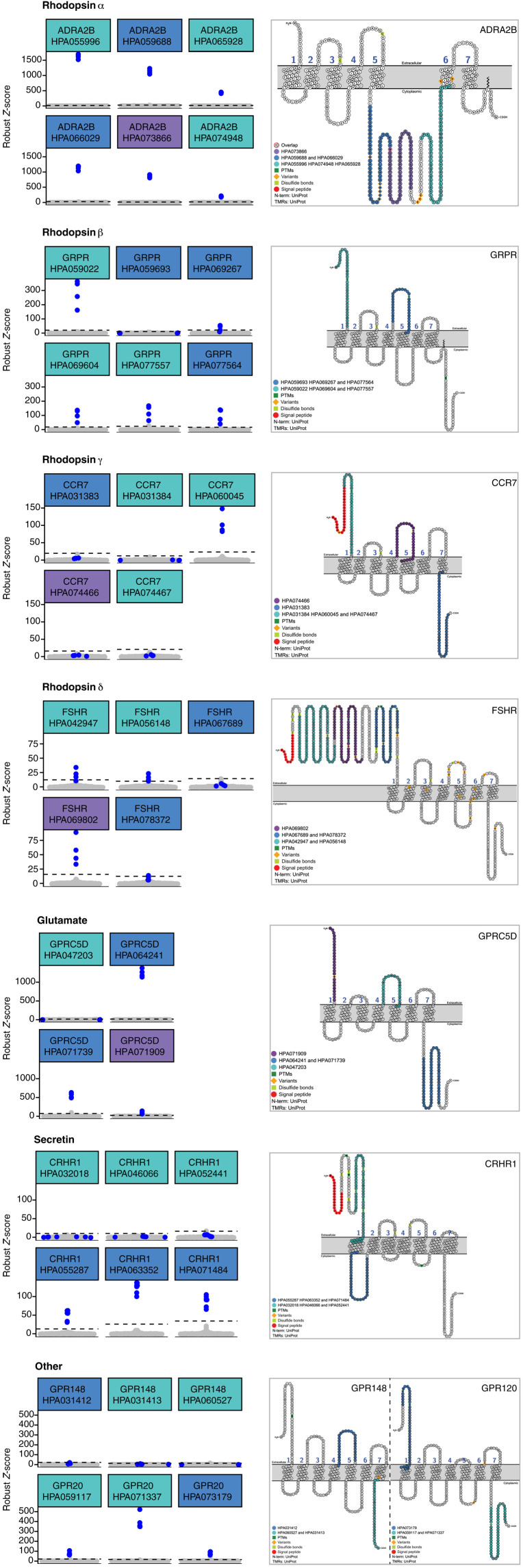
Detection of GPCRs with paired Abs. (Left) Beeswarm plots showing binding events for multiple Abs targeting the same GPCR. Blue dots, intended GPCR; gray dots, unintended GPCRs. Dashed lines correspond to the selectivity cutoff for each HPA Ab. Color coding of HPA Ab ID corresponds to the color coding of the antigen in the snake plot diagram. (Right) Snake plot diagrams showing the antigen sequence used to generate the Ab on the entire protein sequence. Some Abs have the same antigen sequence. Generated with Protter (*37*). Both columns are divided per subfamily.

To exemplify a few scenarios of paired Abs, we selected GPCRs with the highest numbers of paired Abs per respective family. We summarized the performance of each Ab in recognizing solubilized receptors in Fig. 4. For the rhodopsin α subfamily, adrenoceptor α 2B (ADRA2B) was selectively recognized by six of six Abs. They were raised against three distinct epitope regions, all within the intracellular loop 3 (ICL3) of ADRA2B. For the rhodopsin β subfamily, gastrin-releasing peptide receptor was selectively recognized by five of six Abs. The Abs were raised against two distinct epitope regions, one within the extracellular domain (ECD; HPA059693, HPA069267, and HPA077564), and one within the extracellular loop 2 (ECL2; HPA059022, HPA069604, and HPA077557). HPA059693 did not capture the intended target, while the other Abs raised against the same ECD-based antigen, HPA069267 and HPA077564, did selectively capture the target GPCR.

For the δ subfamily, CCR7 was selectively recognized by only one of five Abs, although three distinct antigens were used: one antigen in the ECD, one antigen in ECL2, and one corresponding to the intracellular C-terminal tail of the receptor. HPA060045 was the only Ab tested with selective recognition of CCR7. The other two Abs raised against the same ECD antigen did not detect any target above the threshold. However, the expression levels of CCR7 were lower than most receptors of the δ subfamily and hence classified as “uncertain,” which could indicate that only HPA060045 has a sufficiently high affinity to CCR7 to detect even lower levels of this GPCR. For the rhodopsin γ subfamily, follicle-stimulating hormone receptor (FSHR) was selectively recognized by three of five Abs with less distinct *Z*-score differences than in the previous examples. HPA067689 and HPA078372 were raised against a common antigen close to the first transmembrane helix (TM1) of the N-terminal tail and failed to capture FSHR. The on-target Abs were raised against two antigens further toward the N terminus of the ECD. The three distinct epitope regions are all within the large ECD of FSHR.

Within the glutamate subfamily, GPCR class C group 5 member D (GPRC5D) was selectively recognized by three of four Abs. The failed Ab HPA047203 was raised to the ECL2 region, while the selective Abs targeted its ECD (HPA071909) or intracellular C-terminal tail (HPA064241 and HPA071739). We found cross-reactivity of HPA071739 for the related receptor GPRC5A, albeit at a relatively low R.*Z*-score compared with the R.*Z*-scores for GPRC5D-containing samples. Conversely, the other Ab raised against the same antigen, HPA064241, was highly selective for GPRC5D only. For the secretin-like subfamily, three of six Abs tested for corticotropin-releasing hormone receptor 1 (CRHR1) were on-target. Selective recognition of CRHR1 was linked to an antigen representing the ICL1. Unexpectedly, the three HPA Abs generated against a 60-residue-long antigen on the ECD did not generate Abs to detect solubilized CRHR1 in the assays.

GPCRs grouped as other are represented by GPCR 20 (GPR20) and GPCR 148 (GPR148). For GPR20, all three Abs captured the solubilized receptor, but we observed cross-reactive binding of HPA059117 to the arginine vasopressin receptor 1B (AVPR2) and of HPA073179 to the prolactin-releasing peptide receptor. HPA059117 and the selective Ab HPA071337 were raised against the same intracellular C-terminal tail antigen, while the antigen of HPA073179 is within the ECD and extends slightly into TM1. For the receptor GPR148, none of the three tested Abs recognized the solubilized target GPCR or any other GPCR above the set *Z*-score threshold. The antigens for the Abs are located within the ECL2 and intracellular C-terminal regions of GPR148.

Turning to the results for the receptors in the FZD subfamily (fig. S2), Abs against frizzled receptors 4 and 5 (FZD4 and FZD5) provide examples of differential Ab recognition. Here, one of two Abs tested detected the target proteins, respectively. For FZD4, the selective Ab was raised against a shorter intracellular C-terminal tail antigen (HPA042328) rather than a longer N-terminal one corresponding to the ECD (HPA074833). For FZD5, the Ab targeting the shorter and slightly more N-terminal sequence within the ECD was the selective one (HPA052361), although the other Ab (HPA053811) was also raised against an ECD antigen.

### Deconvolution of off-target binding

**Fig. 5.**
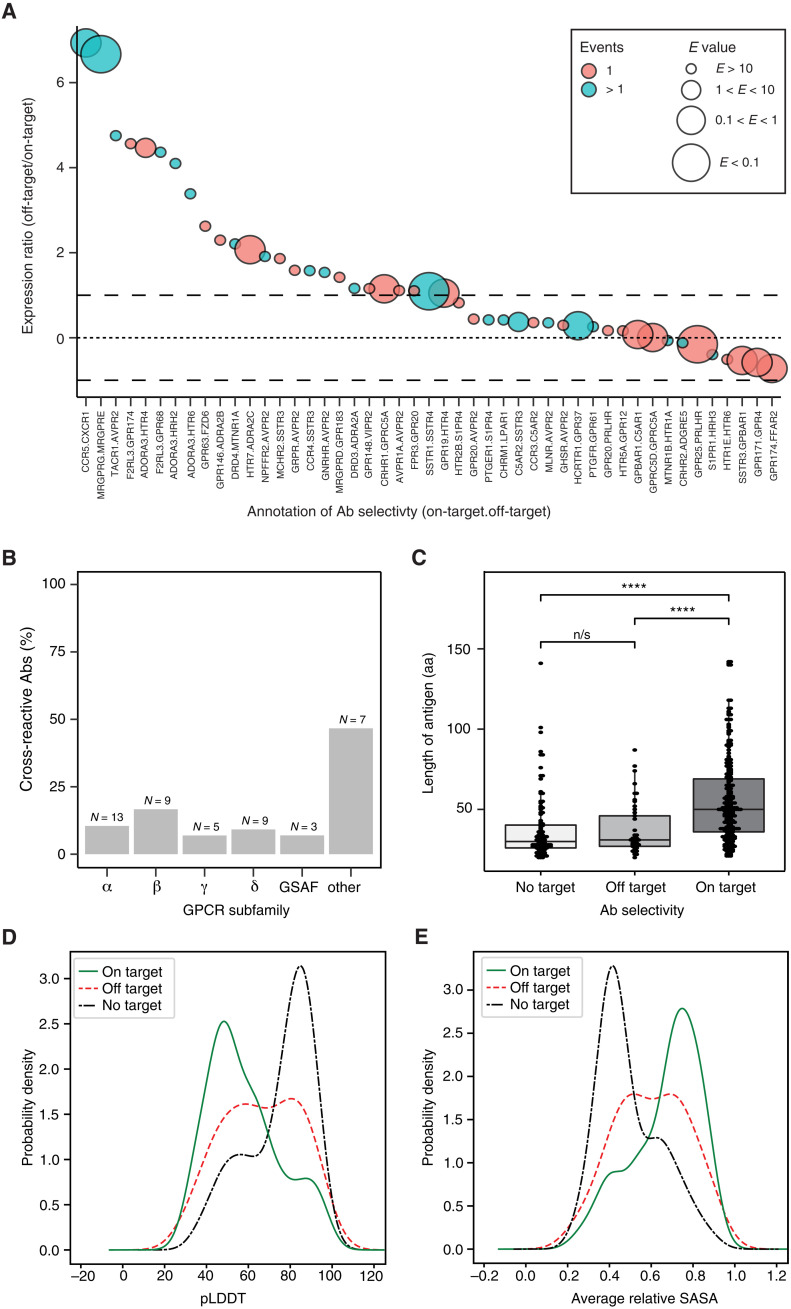
Deconvolution of observed Ab cross-reactivity behavior. (**A**) Deconvoluting off-target binding through expression ratios and sequence homologies. The expression ratio between each on-target GPCR (denominator) and off-target GPCR (numerator) is plotted from highest to lowest. Abs binding off-target GPCRs are listed on the *x* axis according to the format “on-target GPCR.off-target GPCR.” The black dashed line denotes a twofold difference in expression ratio. The dotted line denotes equal on-target and off-target GPCR expression. The size of each circle conveys the *E* value of the target GPCR, off-target GPCR pair. The lower the *E* value, the bigger the circle and the more similar the GPCRs are to each other in the primary structure. Colors indicate whether the off-target GPCR was only captured in one sample (peach) or more than one sample containing it (teal). (**B**) Summary of the number of cross-reacted GPCRs per group tested shown as a percentage of total Abs per GPCR subfamily and as an absolute number. (**C**) Comparison of the antigen length used for producing the HPA Abs binding on-target (*N* = 245) versus those annotated to bind co/off-target (*N* = 45) or no-target (*N* = 109). Significance between the groups was determined by a Kruskal-Wallis test (*P* = 2.5 × 10^−15^). Asterisks represent *P* values from Wilcoxon tests. ****P < 1 × 10^−6^; n/s, not significant. (**D**) The predicted local distance difference test (pLDDT) was conducted for the three Ab selectivity classes (green line, on target; red dashed line, off-target; black dot-dashed line, no target) to determine the levels of confidence in structures predicted for the respective antigens on full-length proteins. (**E**) The averages of relative solvent-accessible surface areas (SASA) were calculated for antigens from the three Ab selectivity classes color coded as in (D) to determine the accessibility of the antigens on the full-length proteins.

A subset of 46 of 407 Abs recognized GPCRs other than the intended targets. To investigate possible reasons for their apparent lack of selectivity, we checked the sequence homologies and differences in expression levels between the on- and off-target GPCR. As shown in Fig. 5, differences in GPCR abundance and sequence similarity contributed to the off-target binding. For approximately half of the Abs, a twofold higher relative abundance of the off-target led to its recognition. For specific cases, lower off-target abundance was compensated by high sequence similarity (*E* < 1; Fig. 5A). We also observed a slight difference in off-target GPCR expression within a subfamily (fig. S3). Overall, there was a similar percentage of Ab cross-reactivity (10 to 20%) for all subfamilies except other (Fig. 5B). The latter group may have a higher rate of off-target Abs because (i) it was the smallest group, (ii) the receptors expressed less sufficiently than other groups (Table 1), (iii) the receptors in this group are of unknown phylogenetic positioning, and, (iv) uniquely, we tested these Abs against all GPCRs in the library. The latter suggests that expanding the number of tested GPCRs likely increases the number of observed off-targets.

Next, we investigated whether any systematic pattern in off-target recognition could be observed (fig. S4). Ten Abs exhibited consistent off-target binding in all replicated samples expressing the off-target GPCR. We observed such examples in five subfamilies. To highlight two examples, the Ab for CCR5 detected the CXCR1 in all samples and the Ab for CRHR2 detected the adhesion GPCR E5 in all samples. Identifying these off-target binders across replicates provides further support and validity for our approach.

The cross-reactivity of most Abs could be attributed to sequence homology or large differences in expression ratio. However, a third category of Abs cross-reacted because of neither abundance nor GPCR sequence homology. We deemed these unselective Abs promiscuous and generally unsuitable for the GPCR analysis in the presented application. We also tested other criteria that might influence whether an Ab bound on-target or not. Considering that all HPA Abs are processed via one pipeline, we checked whether the length of the antigens used to generate the Abs influenced the success rate (Fig. 5C) and found a significant difference (Kruskal-Wallis test, *P =* 2.5 × 10^−13^). The difference between on-target antigens and no-target antigens (Wilcoxon test, *P =* 1.1 × 10^−13^) was more pronounced than between on-target and off-target antigens (Wilcoxon test, *P =* 6.5 × 10^−7^). The antigens of on-target Abs were generally longer (54 amino acids ± 26; *N* = 248) compared with those antigens used to generate Abs that bound off-target or that did not recognize any GPCR (37 amino acids ± 19; *N* = 159).

We next analyzed the predicted structures from AlphaFoldDB (*22*, *23*) of all the antigens to analyze the properties of the epitopes causing Abs to exhibit specific binding or not (table S3). As defined by a low predicted local distance difference test (pLDDT) score, on-target Abs were significantly (*P* = 1.7 × 10^−14^) enriched in protein regions predicted to be disordered (Fig. 5D). Furthermore and as defined by the average relative solvent-accessible surface area, the on-target epitopes were also enhanced (*P =* 1.1 × 10^−15^) in residues exposed to the surrounding (Fig. 5E), enriched in coils (*P =* 3.2 × 10^−9^) and depleted in helices (*P =* 6.6 × 10^−5^) and sheets (*P =* 6.2 × 10^−6^).

### Interactive selectivity analysis

**Fig. 6.**
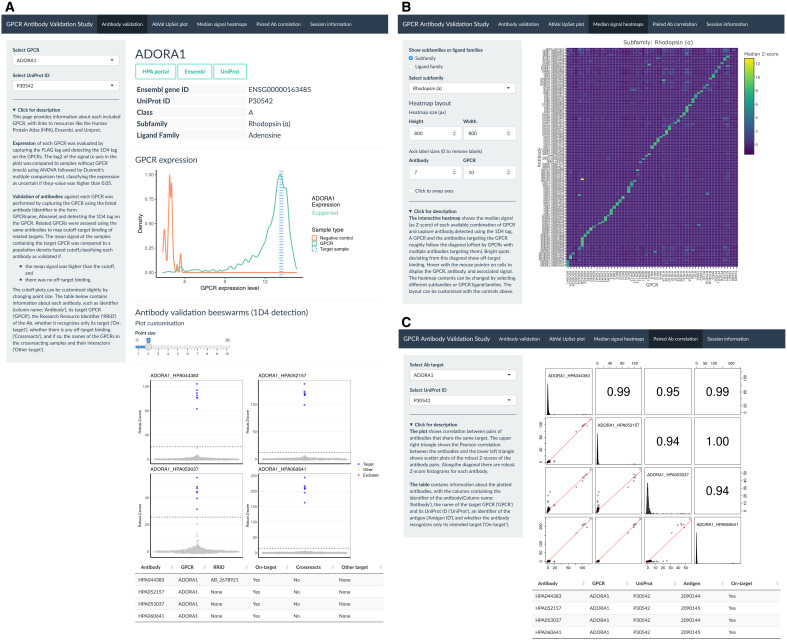
A brief overview of the web-based interface. (**A** to **C**) The interactive web-based interface contains tabs with information about each Ab. (A) The expression of each GPCR is visualized as expression density plots, and the performance of Abs targeting the selected GPCR is visualized as beeswarm plots. (B) Cross-reactivity of Abs with phylogenetically related targets visualized as heatmaps. (C) Comparison of paired Abs. The interface was generated through the shiny R package.

To enable facile access to our data, we developed a web-based interface (Fig. 6). The app can be accessed at https://leod.shinyapps.io/gpcr_abval_2022/, allows interactive Ab- and GPCR-centric browsing of the assay results, and includes information about GPCR expression and enrichment of GPCR per Ab. It presents a summary of the selectivity analysis and allows users to display heatmaps to overview the Ab selectivity per GPCR subfamily. The app also shows correlation analyses of paired Abs raised against a common GPCR.

## DISCUSSION

Most of the Abs used in our study were generated by the HPA project that used a bioinformatic algorithm to select unique features of the primary protein structures to produce highly target-specific antigens (*16*). Newer computational tools have the potential to generate further improvements in antigen designs. The scope of machine learning applications has expanded markedly within the past 3 years and accelerated the prediction of protein structures. The advent of AlphaFold (*22*) has recently been harnessed by others to create in silico models for designing binding reagents for any interface (*24*). It will be interesting to follow the development of such approaches and compare the experimental validation data accompanying computational classification schemes. For the time being, mapping the epitope regions of paired Abs onto two-dimensional GPCR snake diagrams can be complemented by mapping the access to the epitopes in predicted 3D structures. Our analysis highlights that it is unfavorable to generate Abs against regions of antigens that are buried. For example, on the basis of the solved structure of the ECD of CRHR1, it appears that a portion of the antigen sequence corresponding to the three failed anti-CRHR1 Abs (HPA032018, HPA046066, and HPA052441) folds into two β sheets (*25*). The distinct structural features on the GPCR may prevent the Abs raised against antigens mapping to that structural region from recognizing it. The most successful antigens appear to be disordered regions, providing a direction to be further explored for the development of specific anti-GPCR Abs. Nonetheless, the structural prediction of GPCRs and other membrane-bound proteins remains challenging compared to soluble proteins.

Our approach included a systematic assessment of GPCR expression levels. The rhodopsin α GPCR group appears to have the highest percentage of expressed GPCRs and is also the largest group (*N* = 59, 93.2% expressed). In comparison, the rhodopsin δ and other groups had the lowest overall expression (73.9 and 66.7%). The other GPCR group contained only nine receptors, so it may not be a conclusive comparison. The lower overall expression of rhodopsin δ subfamily GPCRs compared with the α subfamily GPCRs may be explained by the prevalence of orphan receptors within the δ group. In our library, 47.8% of the rhodopsin δ subfamily receptors are classified as orphans. In comparison, 13.6% of rhodopsin α subfamily receptors, 4.8% of rhodopsin β subfamily receptors, 3.9% of rhodopsin γ receptors, and 20.7% of GSAF receptors are annotated as orphans. As expected, 100% of the other GPCRs are orphans. Alternative expression schemes offer possible routes to improve expression levels. Still, the low expression yields of orphan receptors across subfamilies may be one of the reasons why endogenous ligands have not yet been identified.

Multiplexed planar protein arrays were used previously to test Ab selectivity (*18*). Recent efforts by Syu *et al.* (*26*) have also resulted in similar arrays for GPCRs. There are, however, key conceptual differences between the planar and SBAs. Crucially, whether the GPCRs or the Abs are immobilized or added in solution differs between the two arrays. Immobilizing and drying the target may alter its molecular conformation and limit the accessibility of some epitopes. In the SBA, micelle-imbedded GPCRs are generated from detergent solubilization, which is highly representative of GPCRs found in cellular membranes. From an analytical perspective, planar and bead arrays follow the ambient analyte assay theory (*27*). For planar arrays, the Abs bind and rebind on the area where a target is immobilized. For the SBA assays, the Abs capture targets that can rebind on the Ab-coupled surface. While Abs are regarded as molecules with a rigid structure, membrane-imbedded, detergent-solubilized GPCRs are known to be structurally labile. Our data suggest that preparing and maintaining the integrity of GPCRs is an essential benefit of the SBA approach. It also enables modulating the GPCR with a higher degree of freedom. Capturing the GPCRs with epitope tags also allows us to ensure that sufficient quantities of each GPCR were present in each assay. This approach also allows for the future use of such GPCRs as targets to assay for circulating auto-Abs in serum samples (*28*, *29*).

We built our approach on pAbs that were readily available for many applications from the HPA project (*30*). Although pAbs have low sustainability due to the limited volumes obtained from the Ab generation process, there are examples where functional mAbs have been produced on antigens identified from pAbs (*31*). The polyepitope characteristic of pAbs may have also contributed to the high success rate observed, as one epitope can compensate for the inaccessibility of another one. The HPA Abs are generated against protein fragments prepared and stored in high-content urea. The fragment length and storage conditions may limit the antigen’s ability to form delicate tertiary structures and represent the native antigen region more accurately. Nonetheless, we found many HPA Abs to recognize the overexpressed GPCRs in micelles, so we wanted to check how well these Abs bound to the endogenous receptors on fixated and denatured tissues or cells. Of the 400 anti-GPCR Abs investigated here, 97 Abs (24%) have been published on the HPA portal (version 21.1). These Abs have been classified as supported or approved by the stringent enhanced validation criteria that focus on the Abs’ utility to stain tissues and cells: Twenty-eight percent (73 of 261) have passed the test for IHC (*9*); twelve percent (46 of 399) have passed for confocal microscopy-based IF (*10*, *11*), and 22% (83 of 370) of the Abs were classified as supportive for the use in immunoblot (URL: https://v21.proteinatlas.org/about/antibody+validation). For an Ab targeting the sphingosine-1-phosphate receptor 4, we observed good agreement between the results of the SBA assay and HPA validation by IF and immunoblot (fig. S5). The HPA Abs have not yet been used systematically in other methods, such as flow cytometry, to determine their performance in detecting membrane proteins under near-native conditions. In general, the differences in the utility of the Abs confirm the influence of assay- and sample-specific conditions and support the need to apply appropriate Ab validation schemes.

The reasons why we observe a 60% success rate in validating the Abs may be explained by a combination of factors. All the mentioned aspects apply equally to the testes with on- or off-target GPCRs. Overexpression of the target reduces the sensitivity burden, as the target becomes the dominant protein, and other proteins may not be able to interfere. Moreover, preparing the samples as membrane fractions, thereby removing nonmembranous but abundant soluble cellular proteins, reduces sample complexity and lowers the selectivity burden for the Abs. Here, we tested each Ab against a subset of GPCRs belonging to one subfamily. These receptors represent the most probable off-targets for a given Ab. Expanding the tests of the receptor libraries to other GPCR subfamilies or other overexpressed membrane proteins would likely increase the number of observed off-target binding events. Using detergent to solubilize the receptors preserves epitope accessibility to facilitate in-solution immunocapture of the GPCRs and circumvents the need to fixate, cross-link, or dry GPCR-containing membranes. In addition, we attribute rebinding events on the Ab-coupled beads as contributing to the validation outcome. Consequently, the chosen assay conditions support the selectivity of the Abs.

Our method of measuring GPCR expression across all samples relies on selective Ab recognition of the FLAG and 1D4 epitope tags engineered into each receptor. FLAG tyrosine sulfation was previously reported to affect GPCR expression and, if present, may result in a “false-negative” determination of GPCR expression (*32*). Although the work by Hunter and colleagues (*32*) focused on a dopamine receptor and all five dopamine receptors in our library expressed well, it is possible that this phenomenon affected other GPCRs that expressed more poorly. Abs validated here are not necessarily specific in other applications. Methods in which solubilization is not possible or where the GPCR epitopes are presented in a different state might require additional validation. We used heterologous overexpression to present high levels of the intended target and all other targets to the bead-bound Abs. Heterologous expression changes the ratios between target abundance over all other proteins in the micelles. It also presents the GPCRs to the Abs at levels that are likely nonphysiological.

Thus far, we have mostly tested HPA Abs, but the approach is not limited to these and can be expanded to other Abs, nanobodies, or scaffold proteins if these can be immobilized to the beads. Caution must be exercised when smaller molecules are used because coupling these to a solid support may limit their functionality. Our approach was built on full-length GPCRs embedded into detergent-lipid micelles. Hence, it does not give us the resolution to determine the exact binding epitopes down to the amino acid level. Using different types and concentrations of detergents and altering the ionic conditions could provide further insights into recognizing specific GPCRs. Tests with varying conditions and combinations thereof could inform about the integrity of a particular epitope and the utility of an Ab in other assays. As of today, we cannot predict a success rate for reproducing selective pAbs and if these pAbs will be functional in the assay even if the antigens that they were raised against have previously generated validated pAbs. A roadmap to develop mAbs can be informed by the results from pAb-based assays: After epitope mapping of pAbs, monospecific Abs can be purified from a polyclonal mix by using the obtained epitopes (*30*). After testing the monospecific fraction in the intended assay, the most suitable epitope can be used to generate mAbs. Nonetheless, the discovery and validation of these Abs will require molecular tests. Preferably, the analytical protocols and assay conditions for the most appropriate applications have already been defined. Last, our work also provides a foundation to identify anti-GPCR auto-Abs associated with a number of disease states in patients (*28*, *33*).

## MATERIALS AND METHODS

### Experimental design

The objective of this study was to determine the selectivity of anti-GPCR Abs. Each binder was tested in an SBA assay against detergent-solubilized, overexpressed GPCR from Expi293F cells. The selectivity of the Abs was tested for their intended GPCR and against GPCRs from the same subfamily. Abs were coupled to color-coded beads, and their binding to a GPCR was detected via fluorescently labeled Abs specific for epitope tags. Per Ab-coupled bead ID, the MFI of at least 32 events was used as data for the selectivity analysis.

### Materials

Information on all Abs used in the SBA generation can be found in table S1. Abs were either from HPA (some of which are commercially available from Atlas Antibodies AB) or purchased from Affinity Biosciences. Research resource identifiers (RRIDs) were stated if available. Expi293F cells were a gift from the Ravetch Lab (The Rockefeller University). Expi293 Expression Medium (catalog no. A1435101) and the ExpiFectamine 293 Transfection Kit (catalog no. A14524) were from Thermo Fisher Scientific. The cells were cultured in 125-ml flasks (catalog no. 431143), 250-ml flasks (catalog no. 431144), and 12-well plates (catalog no. 353043) from Corning. Phycoerythrin (PE)-conjugated anti-FLAG Ab was from BioLegend. The anti-1D4 Ab was conjugated to PE using an Ab conjugation kit from Abcam (catalog no. 102918) according to the manufacturer’s protocols. Half-area 384-well plates were from Greiner. Blocking reagent for enzyme-linked immunosorbent assay (BRE; catalog no. 11112589001) was from Roche. DC Assay kit was from Bio-Rad. Phosphate-buffered saline (PBS) was from Medicago. ProClin 300 (catalog no. 48912-U), cOmplete mini protease inhibitor tablets (catalog no. 11836170001), casein (catalog no. C7078), polyvinyl alcohol (PVA; catalog no. 25213-24-5), polyvinylpyrrolidone (PVP; catalog no. 9003-39-8), and FLAG M2 Ab (catalog no. F3165) were from Sigma-Aldrich. DM (catalog no. D310, CAS 69227-93-6) was from Anatrace. Purified rabbit immunoglobulin G (IgG) was from Bethyl Laboratories (catalog no. P120-101). Anti-mouse IgG- and anti-rabbit IgG-conjugated R-PE were from Jackson ImmunoResearch (catalog nos. 115-116-146 and 111-116-144, respectively).

### Cell culture and transfection

Expi293F were cultured and transfected according to the manufacturer’s instructions. Briefly, cells were cultured in serum-free Expi293 medium using culture flasks under constant shaking at 130 rpm at 37°C with 8% CO_2_. For transfection, cells were counted using a Nexcelom Cellometer Auto T4 and diluted to 2,000,000 cells/ml and were allowed to grow overnight. The next day, the cells were counted and diluted to 3,000,000 cells/ml, and 1.25 ml of cells was transferred to each well of a 12-well culture plate. Transient transfections were then performed with the ExpiFectamine 293 transfection kit (Thermo Fischer Scientific). Each well of cells was transfected with 4 μl of FreeStyle MAX Reagent and 0.25 μg of GPCR plasmid DNA. Total transfected plasmid DNA was kept constant at 1.5 μg per well by adding empty vector pcDNA3.1(+). Enhancers were added 18 to 24 hours after transfection according to the manufacturer’s protocol, and cells were harvested 72 hours after transfection.

### DNA constructs

Epitope-tagged human GPCR DNA constructs were encoded in a pcDNA3.1(+) mammalian expression vector. All GPCRs except CALCRL and FZD4, FZD5, FZD6, and FZD10 relied on the codon-optimized PRESTO-Tango library of signal sequence-FLAG-GPCRs as a starting point to generate 215 FLAG-GPCR-1D4 constructs, with the FLAG tag (DYKDDDDA) following the HA signal sequence MKTIIALSYIFCLVFA. C-terminal components of the original PRESTO-Tango constructs were removed (V2 tail, TEV site, and Tta transcription factor) upon adding 1D4. The amino acid sequence of the C-terminal 1D4 tag is DEASTTVSKTETSQVAPA. The PRESTO-Tango plasmid kit was a gift from B. Roth (Addgene kit no. 1000000068).

All GPCRs retained their endogenous signal sequences, if applicable, in the original PRESTO-Tango library, thereby having two signal sequences in the final plasmid. We removed the endogenous signal sequence for 12 receptors (F2R, GABBR1, GLP1R, GPR156, GPR37, GPR97, GRM1, GRM2, GRM4, GRM5, GRM6, and GRM7), but not from 11 receptors (CALCR, CD97, CRHR1, CRHR2, GCGR, GIPR, GPR114, GPRC5B, GPRC5C, GPRC6A, and VIPR2). FLAG-CALCRL-1D4, with no endogenous signal sequence, was generated by replacing the HA tag with a FLAG tag in the CALCRL construct used previously in Lorenzen *et al*. (*15*).

There were no frizzled receptors in the PRESTO-Tango library, so we included four in-house frizzled receptors. The human frizzled GPCRs FZD4, FZD5, FZD6, and FZD10 cDNAs encode the 23–amino acid residue 5-hydroxytryptamine receptor 3a receptor signal sequence (MALCIPQVLLALFLSMLTGPGEG) in place of the native signal sequence. There is a modified amino acid on position 2 (A instead of R) to optimize the Kozak sequence to GCCGCCACCATGG. An HA tag follows the signal sequence. C-terminal to the receptor is a 1D4 tag. The cDNA for the GPCRs FZD4, FZD5, FZD6, and FZD10 were designed in-house and synthesized through Genewiz.

### Clarified lysate preparation

Cell membranes were solubilized as previously described (*15*). Briefly, cells were solubilized with DM detergent to form micelles around membrane proteins and maintain GPCR structure. Seventy-two hours after transfection, Expi293F cells were harvested and washed twice with cold PBS. Cells were then incubated in solubilization buffer [50 mM Hepes, 1 mM EDTA, 150 mM NaCl, and 5 mM MgCl_2_ (pH 7.4)] with 1% (w/v) DM and cOmplete mini protease inhibitor for 2 hours at 4°C with nutation. Following solubilization, lysates were clarified by centrifugation at 22,000*g* for 20 min at 4°C. Solubilized lysates were then transferred to a microcentrifuge tube, and total protein content was determined by Protein DC assay according to the manufacturer’s specifications. Solubilized lysates were flash-frozen before storage.

### Suspension bead arrays

Anti-GPCR and anti-tag Abs were covalently coupled to color-coded magnetic beads (MagPlex, Luminex Corp.) as previously described (*15*). In short, 1.75 μg of each Ab was diluted in MES buffer [100 mM MES (pH 5.0)] to a final volume of 100 μl. The diluted Abs were then conjugated onto the carboxylated beads using NHS/EDC [N-Hydroxysuccinimide/1-ethyl-3-(3-dimethylaminopropyl) carbodiimide] chemistry. After washing away unbound Abs, the reactions were quenched with BRE buffer overnight. The Ab-coupled beads were subsequently grouped and pooled to form six subfamily-related SBAs. Most of the Abs were rabbit pAbs, and the coupling efficiency was determined using anti-rabbit RPE Abs (Jackson ImmunoResearch). The data were collected using a FLEXMAP 3D instrument (Luminex Corp., xPONENT Software, build 4.3.309.1).

### Assay procedure

The procedure described here is based on and consistent with that of our proof-of-concept study (*15*). Clarified protein lysate was diluted to 2 μg/μl in solubilization buffer (described above) with 0.01% (w/v) DM in a 96-well plate and diluted again 3.6 times in SBA buffer such that 12.5 μl of lysate was combined with 32.5 μl of buffer [PBS containing 0.5% PVA (w/v), 0.8% PVP (w/v), 0.1% (w/v) casein, and 10% rabbit IgG]. We then transferred 45 μl of the solution to a 384-well assay plate containing 5 μl of SBA using CyBio SELMA (Analytik Jena). The lysates and beads were incubated overnight (16 hours) at 4°C. Next, the plates were washed six times with 60 μl of PBS containing 0.05% Tween 20 (PBST) using a BioTek EL406 washer. Detection was enabled by the addition of 50 μl of PE-conjugated anti-tag Abs diluted in BRE containing 0.1% DM, 0.1% Tween 20, and 10% rabbit IgG, and the plate was incubated for 1 hour at 4°C. The final dilution used for the detection Ab (PE-conjugated anti-1D4) was 1:1000. The beads were washed six times with 60 μl of PBST. After the final wash, 60 μl of PBST was added to the beads, and the fluorescence associated with each bead was measured using a Luminex FLEXMAP 3D (Luminex Corp., xPONENT Software, build 4.3.309.1). The data are reported as MFI.

### Web-based interface

A web-based companion R app to the study was made using the shiny package (version 1.7.1) and R version 4.2.0, containerized using Docker (version 20.10.16, build aa7e414) and hosted on the shinyapps.io platform. The Shiny app was created to contain information about each assayed GPCR, such as GPCR expression and on-target and off-target Abs for each, an overview of the validation status of different Abs, interactive heatmaps made using the plotly package (version 4.10.0) showing the reactivity of each Ab against the GPCRs that they were used against, and correlations between pairs of Abs that share the same target using the paired.panels function of the psych package (version 2.2.5). All packages and versions used for visualization are listed within the app.

### Statistical analyses

All data analysis was performed using R version 3.6.0, and plots were produced using the ggplot2 package (version 3.3.6), unless otherwise stated. GPCR expression was evaluated in R using MFI data from FLAG capture and 1D4 detection with significance testing by ordinary one-way analysis of variance (ANOVA) (aov function of the stats package, version 3.6.0), followed by Dunnett’s multiple comparison test to mock (DunnettTest function of the DescTools package, version 0.99.43). Expression per subfamily was tested first, followed by expression of each individual GPCR. GPCRs with a *P*-value below 0.05 were classified as having their expression supported, while other GPCRs were classified as having uncertain expression. The reproducibility of selected GPCRs (ADRB2, GPRC5A, and HCRTR2) was tested in biological triplicates and technical duplicates.

To bring measurements of different GPCRs to a similar scale and identify those Abs that detect only their intended target via detecting the outliers, MFI values were converted to robust *Z*-scores (R.*Z*-score) using the formula {**x** − median(**x**)/[1.4826 × MAD(**x**)]}, where **x** is a vector of measurements from one Ab, calculated separately per Ab.

The selectivity threshold for each anti-GPCR Ab was determined by adding 12 SDs of the expected negative proportion of the population around the Gaussian smoothing population density peak as previously described (*34*). An Ab was classified as on-target if the mean R.*Z*-score of samples containing the target GPCR was higher than the threshold value and if there were no samples with other GPCRs above the threshold. R.*Z*-scores were visualized in beeswarm plots, made using the ggbeeswarm package (version 0.6.0). An overview of the validation was plotted in an UpSet plot showing the distribution of Abs fulfilling different criteria. The plot was made using the ComplexUpset package (version 1.3.3).

Amino acid sequence similarity between antigen sequences and cross-captured proteins was compared using the blast function from the rBLAST package (version 0.99.2) and was reported as *E* values. Alluvial plots were prepared using ggalluvial package (version 0.12.3). The differences in antigen lengths between on-target, off-target, and no-target Abs were compared using a Kruskal-Wallis test, followed by two-tailed Wilcoxon tests between each group.

### Structural mapping

Analysis was performed in Python 3.10.4 using the packages matplotlib and seaborn for visualization. All models of the antigens were downloaded from AlphaFoldDB (*23*) using the corresponding UniProt IDs. The secondary structure and exposed surface area were extracted using the DSSP algorithm (*35*). The disorder of a region was extracted from the pLDDT values, as this is a good indication of disorder (*36*). Statistical differences between the groups were calculated using the independent Student’s *t* test.

## Supplementary Materials

### This PDF file includes:

Figs. S1 to S5 Legends for tables S1 to S3 References

## Notes

View/request a protocol for this paper from *Bio-protocol*.
