# Supplementary material for: Multiplexed selectivity screening of anti-GPCR antibodies
Source: Sci Adv. 2023 May 3;9(18):eadf9297. doi: 10.1126/sciadv.adf9297 (PMC10156119; doi:10.1126/sciadv.adf9297)
Supplement: Supplementary file 1 — Figs. S1 to S5 Legends for tables S1 to S3 References [file sciadv.adf9297_sm.pdf]

Science Advances
AAAS

Supplementary Materials for

Multiplexed selectivity screening of anti-GPCR antibodies

Leo Dahl *et al.*

Corresponding author: Thomas P. Sakmar, sakmar@rockefeller.edu;
Jochen M. Schwenk, jochen.schwenk@scilifelab.se

Sci. Adv. **9**, eadf9297 (2023)
DOI: 10.1126/sciadv.adf9297

The PDF file includes:

Figs. S1 to S5
Legends for tables S1 to S3
References

Other Supplementary Material for this manuscript includes the following:

Tables S1 to S3

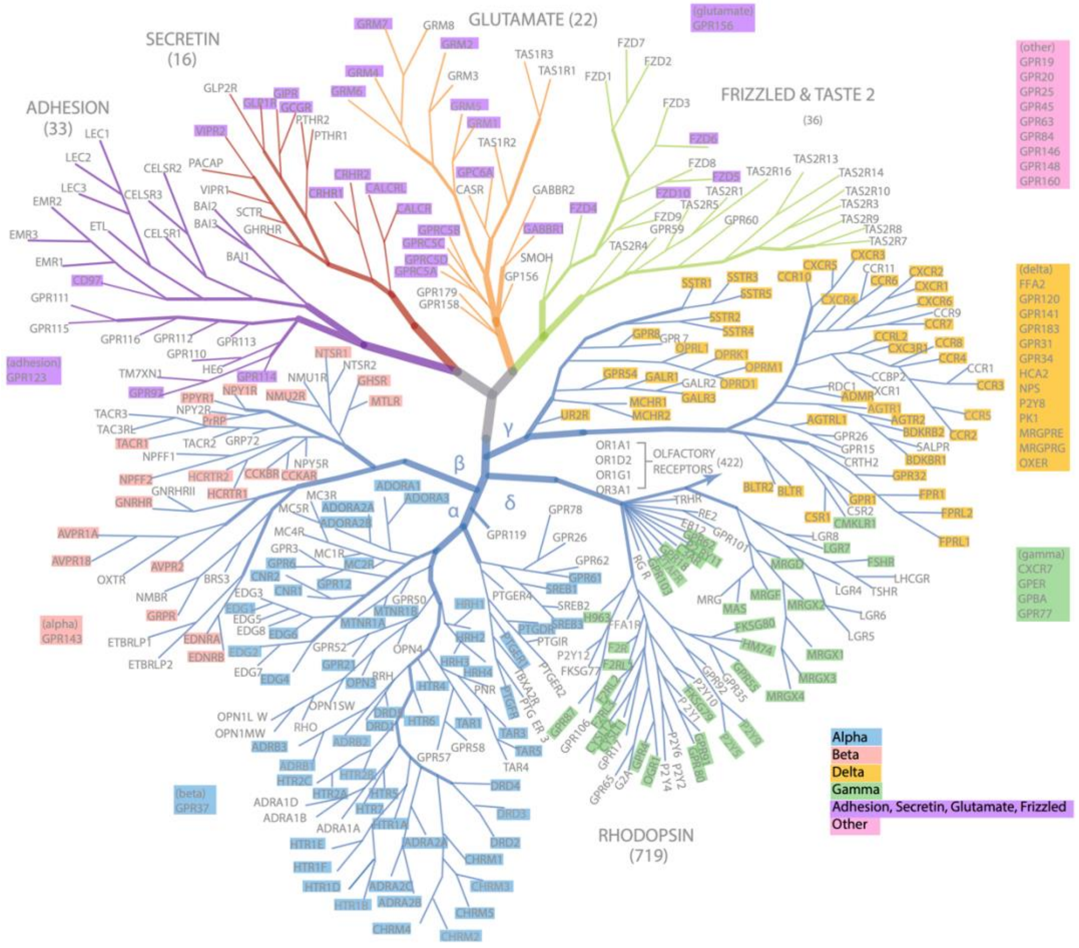

Fig. S1. Positions of selected GPCRs on the phylogenetic tree.

GPCR phylogenetic tree (*2*, *38*) highlighting the 215 receptors used in this study. The color of the highlighting indicates the grouping of the corresponding Abs for each subfamily SBA. Blue, rhodopsin family alpha; peach, rhodopsin family beta; green, rhodopsin family gamma; gold, rhodopsin family delta; purple, Glutamate, Adhesion, Secretin and Frizzed (GSAF) families; pink, other. Adapted from Lv et al. (*39*).

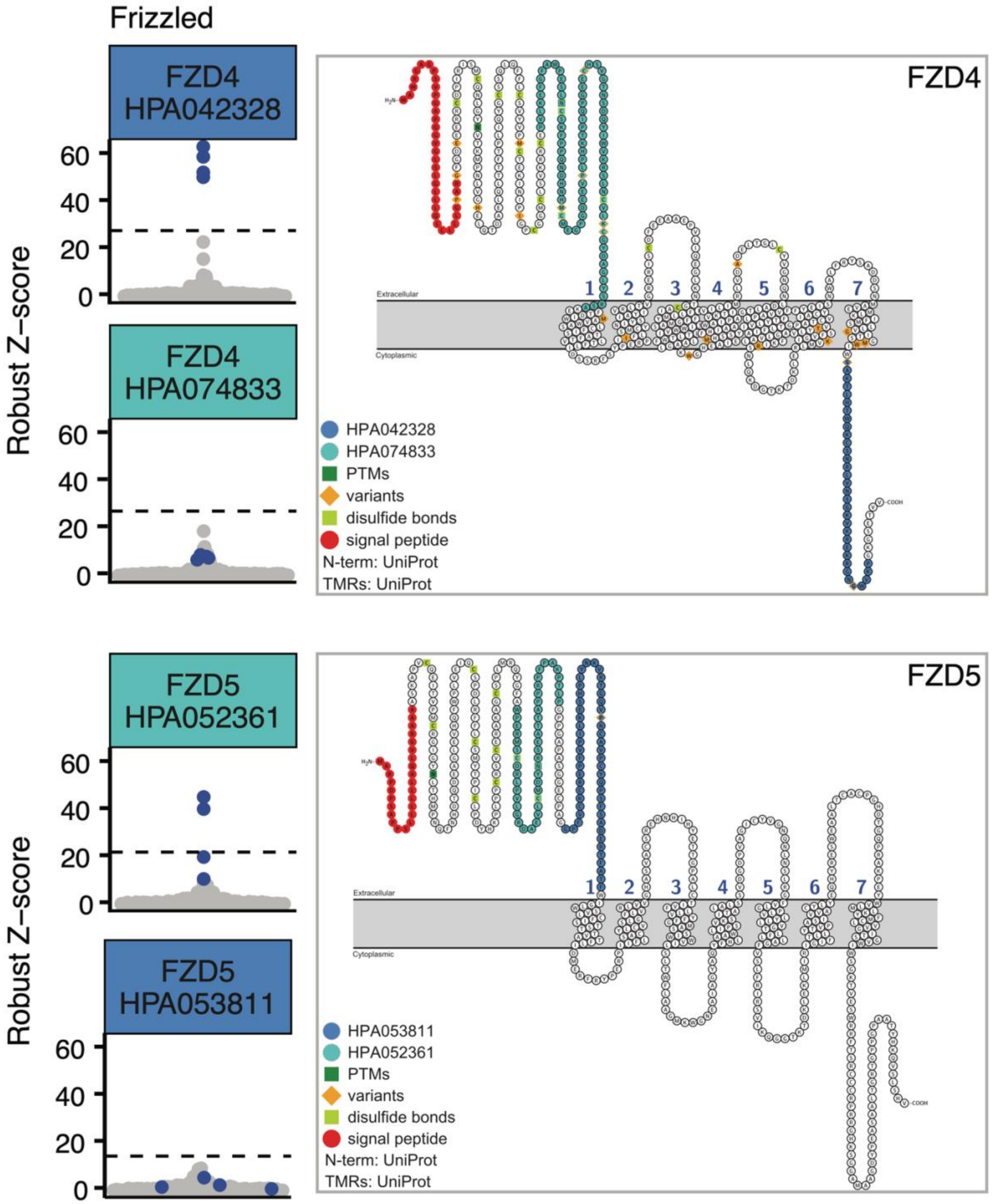

Fig. S2. Detection of FZD GPCRs with paired antibodies.

Left column: Beeswarm plots showing binding events for multiple Abs targeting the same FZD GPCR. Blue dots, intended GPCR; gray dots, unintended GPCRs. Dashed lines correspond to the selectivity cutoff for each HPA Ab. Color coding of HPA Ab ID corresponds to the color coding of the antigen in the snake plot diagram. Right column: Snake plot diagrams showing the antigen sequence used to generate the Ab on the entire protein sequence. Generated with Protter (*37*).

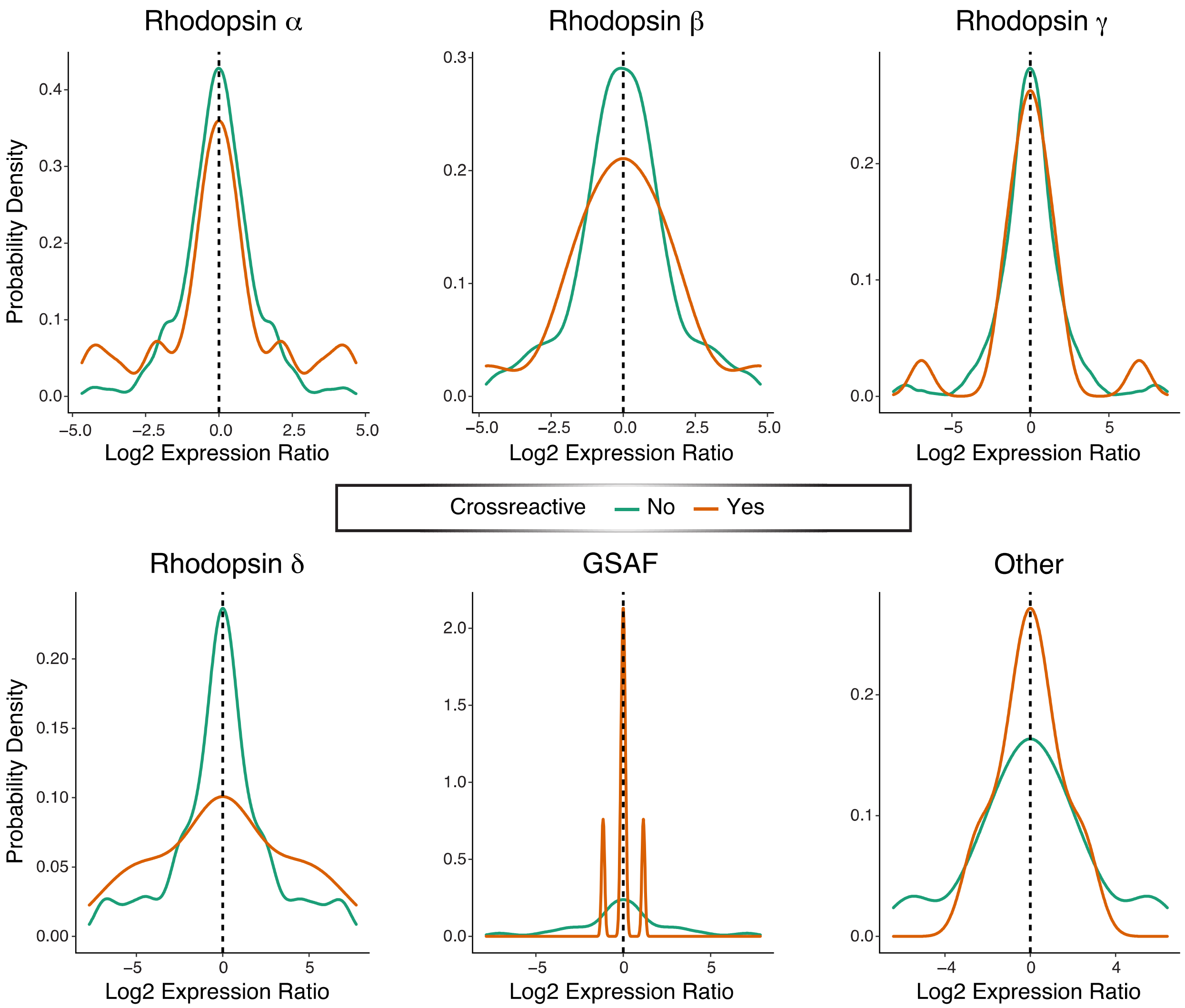

Fig. S3. Expression ratio density plots per GPCR subfamily.

The density plots illustrate the expression ratios between on- and off-target GPCRs for the different subfamilies. For the Abs in the rhodopsin alpha, gamma and delta subfamilies, and glutamate, adhesion, secretin and frizzed (GSAF) subfamilies, there was a higher abundance of off-target GPCRs (orange) compared with on-target GPCRs (green).

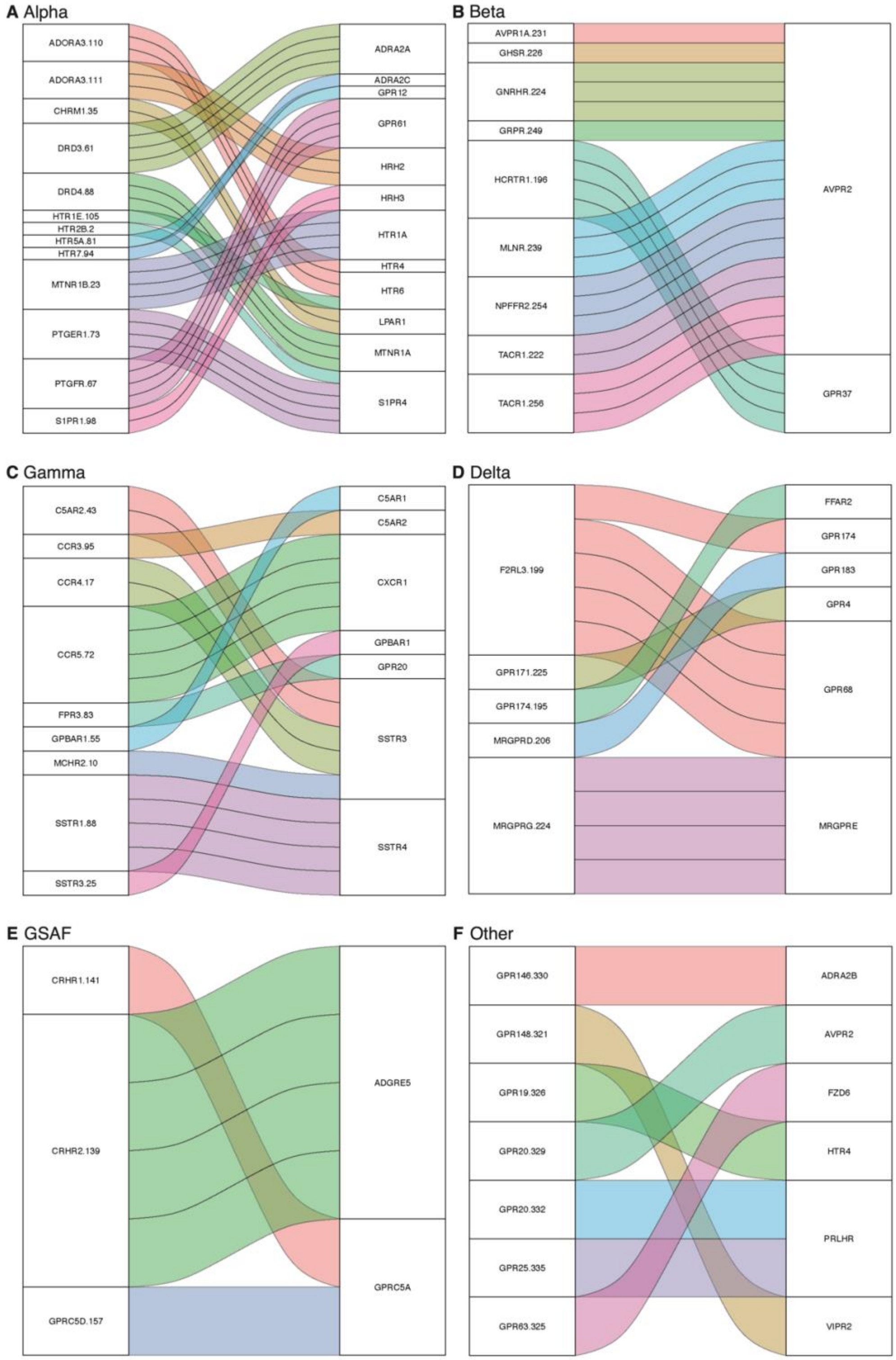

Fig. S4. Summary of antibody cross-reactivity per phylogenetic group.

(A-F) Alluvial plots per GPCR subfamily (**A-D**, Rhodopsin alpha, beta, gamma and delta; **E**, Glutamate, Secretin, Adhesion, Frizzled (GSAF); **F**, other) showing Abs on the left axis and their off-target GPCRs on the right axis. Axes are sorted alphabetically. Each line represents one GPCR-containing sample assayed with the Ab. The connections are colored by Ab.

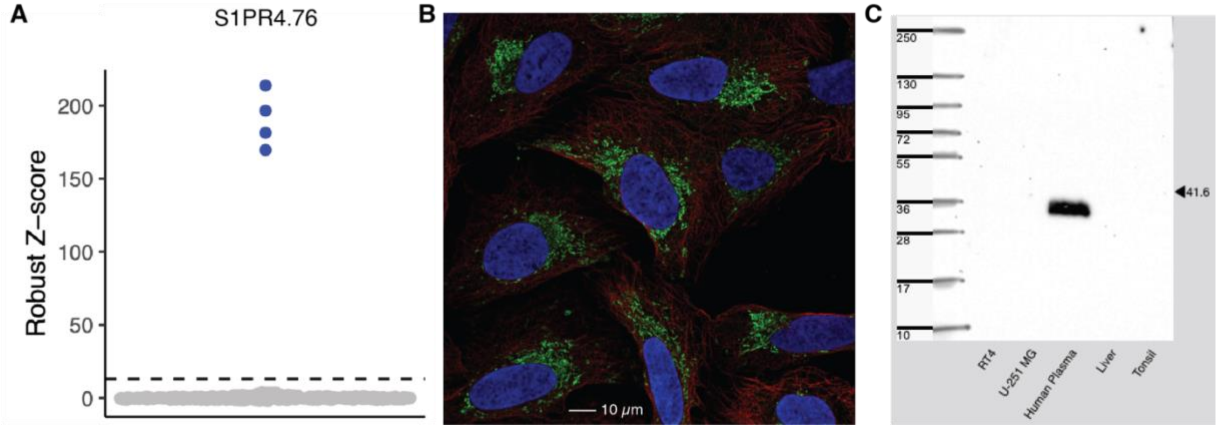

Fig. S5. Utility of tested antibodies in other assays.

(A) An example of an on-target Ab used in other assays. The Ab HPA067232 selectively recognized the GPCR Sphingosine-1-phosphate receptor 4 (SP1R4) as validated by SBA. The results of the SBA assay are in line with the orthogonal validation performed by the HPA to localize SP1R4 to the mitochondria with immunofluorescence analysis of U-2 OS cells **(B)** and by immunoblot **(C)**. Images from **(B)** and **(C)** are from the Human Protein Atlas [URL: v22.proteinatlas.org/ENSG00000125910-S1PR4/summary/antibody].

Supplementary tables

Tables are provided as separate files, and their legends can be found below.

Table S1. HPA antibodies and GPCRs. List of all used HPA Abs, their target GPCRs, and information about each GPCR and antigen. The performance of each Ab in the validation is also specified.

Table S2. GPCR expression test results per subfamily, per GPCR and for replicates of three select GPCRs.

Table S3. Structural predictions of antigens.

REFERENCES AND NOTES

1. R. Santos, O. Ursu, A. Gaulton, A. P. Bento, R. S. Donadi, C. G. Bologa, A. Karlsson, B. al-Lazikani, A. Hersey, T. I. Oprea, J. P. Overington, A comprehensive map of molecular drug targets. *Nat. Rev. Drug Discov.* **16**, 19–34 (2017).

2. R. C. Stevens, V. Cherezov, V. Katritch, R. Abagyan, P. Kuhn, H. Rosen, K. Wüthrich, The GPCR Network: A large-scale collaboration to determine human GPCR structure and function. *Nat. Rev. Drug Discov.* **12**, 25–34 (2013).

3. S. Li, H. Luo, R. Lou, C. Tian, C. Miao, L. Xia, C. Pan, X. Duan, T. Dang, H. Li, C. Fan, P. Tang, Z. Zhang, Y. Liu, Y. Li, F. Xu, Y. Zhang, G. Zhong, J. Hu, W. Shui, Multiregional profiling of the brain transmembrane proteome uncovers novel regulators of depression. *Sci. Adv.* **7**, eabf0634 (2021).

4. M. Baker, Reproducibility crisis: Blame it on the antibodies. *Nature* **521**, 274–276 (2015).

5. A. Bradbury, A. Pluckthun, Reproducibility: Standardize antibodies used in research. *Nature* **518**, 27–29 (2015).

6. M. Uhlen, A. Bandrowski, S. Carr, A. Edwards, J. Ellenberg, E. Lundberg, D. L. Rimm, H. Rodriguez, T. Hiltke, M. Snyder, T. Yamamoto, A proposal for validation of antibodies. *Nat. Methods* **13**, 823–827 (2016).

7. A. E. Bandrowski, M. E. Martone, RRIDs: A simple step toward improving reproducibility through rigor and transparency of experimental methods. *Neuron* **90**, 434–436 (2016).

8. C. Fredolini, S. Byström, E. Pin, F. Edfors, D. Tamburro, M. J. Iglesias, A. Häggmark, M.G. Hong, M. Uhlen, P. Nilsson, J. M. Schwenk, Immunocapture strategies in translational proteomics. *Expert Rev. Proteomics* **13**, 83–98 (2016).

9. A. Sivertsson, E. Lindström, P. Oksvold, B. Katona, F. Hikmet, J. Vuu, J. Gustavsson, E. Sjöstedt, K. von Feilitzen, C. Kampf, J.M. Schwenk, M. Uhlén, C. Lindskog, Enhanced

validation of antibodies enables the discovery of missing proteins. *J. Proteome Res.* **19**, 4766–4781 (2020).

10. M. Skogs, C. Stadler, R. Schutten, M. Hjelmare, C. Gnann, L. Björk, I. Poser, A. Hyman, M. Uhlén, E. Lundberg, Antibody validation in bioimaging applications based on endogenous expression of tagged proteins. *J. Proteome Res.* **16**, 147–155 (2017).

11. C. Stadler, M. Hjelmare, B. Neumann, K. Jonasson, R. Pepperkok, M. Uhlén, E. Lundberg, Systematic validation of antibody binding and protein subcellular localization using siRNA and confocal microscopy. *J. Proteomics* **75**, 2236–2251 (2012).

12. K. Sikorski, A. Mehta, M. Inngjerdingen, F. Thakor, S. Kling, T. Kalina, T. A. Nyman, M. E. Stensland, W. Zhou, G. A. de Souza, L. Holden, J. Stuchly, M. Templin, F. Lund-Johansen, A high-throughput pipeline for validation of antibodies. *Nat. Methods* **15**, 909–912 (2018).

13. A. Tanaka, H. Matsuoka, H. Nishino, J. Imanishi, Antiherpetic action of prostaglandin D2. *Prostaglandins Leukot. Med.* **25**, 131–138 (1986).

14. C. Fredolini, S. Byström, L. Sanchez-Rivera, M. Ioannou, D. Tamburro, F. Pontén, R. M. Branca, P. Nilsson, J. Lehtiö, J. M. Schwenk, Systematic assessment of antibody selectivity in plasma based on a resource of enrichment profiles. *Sci. Rep.* **9**, 8324 (2019).

15. E. Lorenzen, T. Dodig-Crnković, I. B. Kotliar, E. Pin, E. Ceraudo, R. D. Vaughan, M. Uhlèn, T. Huber, J. M. Schwenk, T. P. Sakmar, Multiplexed analysis of the secretin-like GPCR-RAMP interactome. *Sci. Adv.* **5**, eaaw2778 (2019).

16. L. Berglund, E. Björling, K. Jonasson, J. Rockberg, L. Fagerberg, C. al-Khalili Szigyarto, Å. Sivertsson, M. Uhlén, A whole-genome bioinformatics approach to selection of antigens for systematic antibody generation. *Proteomics* **8**, 2832–2839 (2008).

17. P. Nilsson, L. Paavilainen, K. Larsson, J. Ödling, M. Sundberg, A.C. Andersson, C. Kampf, A. Persson, C.A.K. Szigyarto, J. Ottosson, E. Björling, S. Hober, H. Wernérus, K. Wester, F. Pontén, M. Uhlen, Towards a human proteome atlas: High-throughput generation of mono-specific antibodies for tissue profiling. *Proteomics* **5**, 4327–4337 (2005).

18. R. Sjoberg, C. Mattsson, E. Andersson, C. Hellström, M. Uhlen, J. M. Schwenk, B. Ayoglu, P. Nilsson, Exploration of high-density protein microarrays for antibody validation and autoimmunity profiling. *N. Biotechnol.* **33**, 582–592 (2016).

19. C. Algenas, C. Agaton, L. Fagerberg, A. Asplund, L. Björling, E. Björling, C. Kampf, E. Lundberg, P. Nilsson, A. Persson, K. Wester, F. Pontén, H. Wernérus, M. Uhlén, J. O. Takanen, S. Hober, Antibody performance in western blot applications is context-dependent. *Biotechnol. J.* **9**, 435–445 (2014).

20. P. J. Thul, L. Åkesson, M. Wiking, D. Mahdessian, A. Geladaki, H. Ait Blal, T. Alm, A. Asplund, L. Björk, L. M. Breckels, A. Bäckström, F. Danielsson, L. Fagerberg, J. Fall, L. Gatto, C. Gnann, S. Hober, M. Hjelmare, F. Johansson, S. Lee, C. Lindskog, J. Mulder, C. M. Mulvey, P. Nilsson, P. Oksvold, J. Rockberg, R. Schutten, J. M. Schwenk, Å. Sivertsson, E. Sjöstedt, M. Skogs, C. Stadler, D. P. Sullivan, H. Tegel, C. Winsnes, C. Zhang, M. Zwahlen, A. Mardinoglu, F. Pontén, K. von Feilitzen, K. S. Lilley, M. Uhlén, E. Lundberg, A subcellular map of the human proteome. *Science* **356**, (2017).

21. M. Uhlen, L. Fagerberg, B. M. Hallström, C. Lindskog, P. Oksvold, A. Mardinoglu, Å. Sivertsson, C. Kampf, E. Sjöstedt, A. Asplund, I. M. Olsson, K. Edlund, E. Lundberg, S. Navani, C. A.-K. Szigyarto, J. Odeberg, D. Djureinovic, J. O. Takanen, S. Hober, T. Alm, P.-H. Edqvist, H. Berling, H. Tegel, J. Mulder, J. Rockberg, P. Nilsson, J. M. Schwenk, M. Hamsten, K. von Feilitzen, M. Forsberg, L. Persson, F. Johansson, M. Zwahlen, G. von Heijne, J. Nielsen, F. Pontén, Proteomics. Tissue-based map of the human proteome. *Science* **347**, 1260419 (2015).

22. J. Jumper, R. Evans, A. Pritzel, T. Green, M. Figurnov, O. Ronneberger, K. Tunyasuvunakool, R. Bates, A. Žídek, A. Potapenko, A. Bridgland, C. Meyer, S. A. A. Kohl, A. J. Ballard, A. Cowie, B. Romera-Paredes, S. Nikolov, R. Jain, J. Adler, T. Back, S. Petersen, D. Reiman, E. Clancy, M. Zielinski, M. Steinegger, M. Pacholska, T. Berghammer, S. Bodenstein, D. Silver, O. Vinyals, A. W. Senior, K. Kavukcuoglu, P. Kohli, D. Hassabis, Highly accurate protein structure prediction with AlphaFold. *Nature* **596**, 583–589 (2021).

23. M. Varadi, S. Anyango, M. Deshpande, S. Nair, C. Natassia, G. Yordanova, D. Yuan, O. Stroe, G. Wood, A. Laydon, A. Žídek, T. Green, K. Tunyasuvunakool, S. Petersen, J. Jumper, E. Clancy, R. Green, A. Vora, M. Lutfi, M. Figurnov, A. Cowie, N. Hobbs, P. Kohli, G. Kleywegt, E. Birney, D. Hassabis, S. Velankar, AlphaFold Protein Structure Database: Massively expanding the structural coverage of protein-sequence space with high-accuracy models. *Nucleic Acids Res.* **50**, D439-D444 (2022).

24. P. Bryant, A. Elofsson, EvoBind: In silico directed evolution of peptide binders with AlphaFold. *bioRxiv*, 2022.07.23.501214 (2022).

25. K. Pal, K. Swaminathan, H. E. Xu, A. A. Pioszak, Structural basis for hormone recognition by the human CRFR2α G protein-coupled receptor. *J. Biol. Chem.* **285**, 40351–40361 (2010).

26. G. D. Syu, S. C. Wang, G. Ma, S. Liu, D. Pearce, A. Prakash, B. Henson, L. C. Weng, D. Ghosh, P. Ramos, D. Eichinger, I. Pino, X. Dong, J. Xiao, S. Wang, N. Tao, K. S. Kim, P. J. Desai, H. Zhu, Development and application of a high-content virion display human GPCR array. *Nat. Commun.* **10**, 1997 (2019).

27. R. Ekins, F. Chu, E. Biggart, Multispot, multianalyte, immunoassay. *Ann. Biol. Clin.* **48**, 655–666 (1990).

28. O. Cabral-Marques, G. Halpert, L. F. Schimke, Y. Ostrinski, A. Vojdani, G. C. Baiocchi, P. P. Freire, I. S. Filgueiras, I. Zyskind, M. T. Lattin, F. Tran, S. Schreiber, A. H. C. Marques, D. R. Plaça, D. L. M. Fonseca, J. Y. Humrich, A. Müller, L. M. Giil, H. Graßhoff, A. Schumann, A. Hackel, J. Junker, C. Meyer, H. D. Ochs, Y. B. Lavi, C. Scheibenbogen, R. Dechend, I. Jurisica, K. Schulze-Forster, J. I. Silverberg, H. Amital, J. Zimmerman, H. Heidecke, A. Z. Rosenberg, G. Riemekasten, Y. Shoenfeld, Autoantibodies targeting GPCRs and RAS-related molecules associate with COVID-19 severity. *Nat. Commun.* **13**, 1220 (2022).

29. G. Riemekasten, F. Petersen, H. Heidecke, What makes antibodies against G protein-coupled receptors so special? A novel concept to understand chronic diseases. *Front. Immunol.* **11**, 564526 (2020).

30. B. Hjelm, B. Forsström, U. Igel, H. Johannesson, C. Stadler, E. Lundberg, F. Ponten, A. Sjöberg, J. Rockberg, J. M. Schwenk, P. Nilsson, C. Johansson, M. Uhlén, Generation of monospecific antibodies based on affinity capture of polyclonal antibodies. *Protein Sci.* **20**, 1824–1835 (2011).

31. U. Qundos, H. Johannesson, C. Fredolini, G. O'Hurley, R. Branca, M. Uhlén, F. Wiklund, A. Bjartell, P. Nilsson, J. M. Schwenk, Analysis of plasma from prostate cancer patients links decreased carnosine dipeptidase 1 levels to lymph node metastasis. *Transl. Proteom.* **2**, 14–24 (2014).

32. M. R. Hunter, N. L. Grimsey, M. Glass, Sulfation of the FLAG epitope is affected by co-expression of G protein-coupled receptors in a mammalian cell model. *Sci. Rep.* **6**, 27316 (2016).

33. M. A. Skiba, A. C. Kruse, Autoantibodies as endogenous modulators of GPCR signaling. *Trends Pharmacol. Sci.* **42**, 135–150 (2021).

34. N. Roxhed, A. Bendes, M. Dale, C. Mattsson, L. Hanke, T. Dodig-Crnković, M. Christian, B. Meineke, S. Elsässer, J. Andréll, S. Havervall, C. Thålin, C. Eklund, J. Dillner, O. Beck, C. E. Thomas, G. McInerney, M. G. Hong, B. Murrell, C. Fredolini, J. M. Schwenk, Multianalyte serology in home-sampled blood enables an unbiased assessment of the immune response against SARS-CoV-2. *Nat. Commun.* **12**, 3695 (2021).

35. W. Kabsch, C. Sander, Dictionary of protein secondary structure: pattern recognition of hydrogen-bonded and geometrical features. *Biopolymers* **22**, 2577–2637 (1983).

36. J. L. Binder, J. Berendzen, A. O. Stevens, Y. He, J. Wang, N. V. Dokholyan, T. I. Oprea, AlphaFold illuminates half of the dark human proteins. *Curr. Opin. Struct. Biol.* **74**, 102372 (2022).

37. U. Omasits, C. H. Ahrens, S. Muller, B. Wollscheid, Protter: Interactive protein feature visualization and integration with experimental proteomic data. *Bioinformatics* **30**, 884–886 (2014).

38. R. Fredriksson, M. C. Lagerstrom, L. G. Lundin, H. B. Schioth, The G-protein-coupled receptors in the human genome form five main families. Phylogenetic analysis, paralogon groups, and fingerprints. *Mol. Pharmacol.* **63**, 1256–1272 (2003).

39. X. Lv, J. Liu, Q. Shi, Q. Tan, D. Wu, J. J. Skinner, A. L. Walker, L. Zhao, X. Gu, N. Chen, L. Xue, P. Si, L. Zhang, Z. Wang, V. Katritch, Z. J. Liu, R. C. Stevens, In vitro expression and analysis of the 826 human G protein-coupled receptors. *Protein Cell* **7**, 325–337 (2016).
